# The complete mitochondrial genome of *Plautia crossota* (Hemiptera: Pentatomidae)

**DOI:** 10.1080/23802359.2019.1627924

**Published:** 2019-07-11

**Authors:** Yeying Wang, Yubao Duan, Xiaofei Yang

**Affiliations:** a Key Laboratory of State Forestry Administration on Biodiversity Conservation in Karst Mountainous Area of Southwestern of China, Guizhou Normal University, Guiyang, P. R. China;; b Key Laboratory for Forest Resources Conservation and Utilization in the Southwest Mountains of China, Ministry of Education, Southwest Forestry University, Kunming, P. R. China;; c College of Tea Science, Guizhou University, Guiyang, P. R. China

**Keywords:** Mitochondrial genome, phylogeny, *Plautia crossota*, Hemiptera

## Abstract

In this study, we elucidated the complete mitochondrial genome (mitogenome) of *Plautia crossota*. The circular mitogenome is 16,809 bp long, including 13 protein-coding genes, 22 tRNA genes, 2 rRNA genes, and a non-coding control region. The overall base composition is as follows: A, 41.6%; T, 32.75%; C, 14.84%; G, 10.81%; a slight A + T bias of 74.35%. Phylogenetic analysis of 20 species of Pentatomoidea revealed that *Plautia crossota* was closer to *Nezara viridula*.

*Plautia crossota*, tribe Antestiini of the subfamily Pentatominae, is widely distributed in Africa and southeast of Asia (Rider et al. 2002). In China, it is widespread, occurring in Hubei, Hunan, Fujian, Sichuan, Guizhou, Yunnan, Guangxi, Guangdong, Hainan, and Tibet. The earliest name was *Cimex fimbriatus* Fabricius, which was defined as type species when the genus *Plautia* Stal, 1865 was established first, but it was recognized as a preoccupied name in 1909 (Liu and Zheng 1994). The verification of type specimen of *fimbriatus* Fabr. confirmed that the two names are indeed synonyms (Rider et al. 2002). But even so, the existing recordings on ‘*crossota*’ and ‘*fimbriata*’ around the world are still not clear. A mitochondrial genome is an effective tool for species identification (Galtier et al. 2009). Therefore, the complete mitochondrial genome of *P. crossota* and its phylogenetic relationships within Pentatomoidea were investigated in this study. The results of this study may contribute to available identification and further phylogenetic analyses of the members of family Pentatomidae.

This was the first complete plastome sequence from the genus *Plautia* Stal, 1865. Specimens of *P. crossota* were collected from Yingge Mountain Nature Reserves in Hainan, China (19°02′54.8″N, 109°34′08.7″E; alt. 600 m). The voucher specimens of the species and the isolated DNA were stored in the Guizhou Normal University.

Genomic DNA isolated was sequenced using Illumina’s Miseq (Illumina, San Diego, CA). The resultant reads were assembled and annotated using the A5-miseq v20150522 (Coil et al. 2015) and SPAdesv3.9.0 (Nurk et al. 2013). The complete mitochondrial genome was annotated with MITOS (http://mitos.bioinf.uni-leipzig.de/) (Bernt et al. 2013). Phylogenetic analysis was carried out on the basis of 20 available mitogenomes of Pentatomoidea insects in GenBank including newly sequenced mitogenome of *P. crossota* with maximum-likelihood (ML) methods using MEGA v7.0.14 (Kumar et al. 2016).

A total of 1.3G bp reads in fastq format were obtained and subjected to mitochondrial genome assembly. The *P. crossota* mitochondrial genome forms a closed loop that is 16,809 bp long (accession number MK757497). It comprises of 13 protein-coding genes (PCGs), 22 tRNA genes, 2 rRNA genes, and a control region (D-loop). The overall base composition of the mitogenome is the following: A, 41.6%; T, 32.75%; C, 14.84%; G, 10.81%; a slight A + T bias of 74.35%.

Phylogenetic analysis of *P. crossota* with other 19 species of Pentatomoidea was performed using the sequences of their complete mitogenome (Figure 1). *Plautia crossota* was observed to be closer to *Nezara viridula* phylogenetically among studied species of Pentatomoidea. The present dataset may potentially contribute to taxonomic and phylogenetic studies of the Hemiptera.

**Figure 1.**
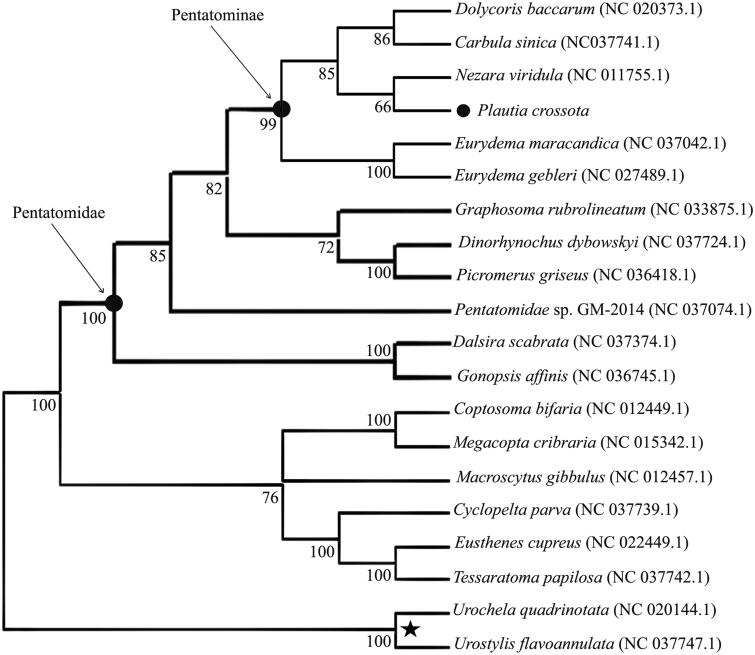
Phylogenetic relationships of 20 species of superfamily Pentatomoidea based on their complete mitochondrial genomes, determined using the neighbour-joining method. Bootstrap support values (1000 replicates) are indicated at the nodes. The number after the species name is the GenBank accession number. Five-pointed star indicates the outgroup.
